# T2 heterogeneity as an *in vivo* marker of microstructural integrity in medial temporal lobe subfields in ageing and mild cognitive impairment

**DOI:** 10.1016/j.neuroimage.2021.118214

**Published:** 2021-09

**Authors:** Alfie R. Wearn, Volkan Nurdal, Esther Saunders-Jennings, Michael J. Knight, Christopher R. Madan, Sean-James Fallon, Hanna K. Isotalus, Risto A. Kauppinen, Elizabeth J. Coulthard

**Affiliations:** a Bristol Medical School, University of Bristol, Institute of Clinical Neurosciences, Learning & Research Building at Southmead Hospital, Bristol BS10 5NB, UK; b School of Psychological Science, University of Bristol, Bristol, UK; c Clinical Neurosciences, North Bristol NHS Trust, Bristol, UK; d School of Psychology, University of Nottingham, Nottingham, UK; e National Institute for Health Research Bristol Biomedical Research Centre, University Hospitals Bristol, NHS Foundation Trust and University of Bristol, Bristol, UK; f Faculty of Engineering, University of Bristol, Bristol, UK

**Keywords:** Magnetic resonance imaging, Alzheimer's disease, Early diagnosis, Ageing, Hippocampal subfields, Medial temporal lobe, T2 relaxometry, T2 heterogeneity

## Abstract

A better understanding of early brain changes that precede loss of independence in diseases like Alzheimer's disease (AD) is critical for development of disease-modifying therapies. Quantitative MRI, such as T2 relaxometry, can identify microstructural changes relevant to early stages of pathology. Recent evidence suggests heterogeneity of T2 may be a more informative MRI measure of early pathology than absolute T2. Here we test whether T2 markers of brain integrity precede the volume changes we know are present in established AD and whether such changes are most marked in medial temporal lobe (MTL) subfields known to be most affected early in AD. We show that T2 heterogeneity was greater in people with mild cognitive impairment (MCI; *n* = 49) compared to healthy older controls (*n* = 99) in all MTL subfields, but this increase was greatest in MTL cortices, and smallest in dentate gyrus. This reflects the spatio-temporal progression of neurodegeneration in AD. T2 heterogeneity in CA1-3 and entorhinal cortex and volume of entorhinal cortex showed some ability to predict cognitive decline, where absolute T2 could not, however further studies are required to verify this result. Increases in T2 heterogeneity in MTL cortices may reflect localised pathological change and may present as one of the earliest detectible brain changes prior to atrophy. Finally, we describe a mechanism by which memory, as measured by accuracy and reaction time on a paired associate learning task, deteriorates with age. Age-related memory deficits were explained in part by lower subfield volumes, which in turn were directly associated with greater T2 heterogeneity. We propose that tissue with high T2 heterogeneity represents extant tissue at risk of permanent damage but with the potential for therapeutic rescue. This has implications for early detection of neurodegenerative diseases and the study of brain-behaviour relationships.

## 1 Introduction

Accurate early diagnosis of Alzheimer's disease (AD) is likely a necessity for development of disease-modifying therapies (Cummings et al., 2014; Alzheimer's Association, 2015). Manifestation of cognitive symptoms, although required for clinical diagnosis, is a relatively late stage in the pathological process (Jack et al., 2010). Thus, clinical interventions after the appearance of cognitive deficits may be too late to restore brain health. A better understanding of the brain changes that precede loss of daily independence will help design early markers.

Structural and quantitative MRI show promise in their ability to identify changes in the brain that indicate early Alzheimer's pathology. Identifying which people with mild cognitive impairment (MCI) will progress to AD dementia has been shown to be possible by measuring the volume of subfields within the medial temporal lobe (MTL) (Chételat et al., 2008; de Flores et al., 2015a, 2015b; deToledo-Morrell et al., 2004; Apostolova et al., 2006, 2010). In these groups, large changes in volume tend to indicate significant, and likely irreversible, atrophy. Smaller scale microstructural changes that occur prior to volume loss could help to identify patients in which such a treatment can still rescue damaged brain tissue.

Recently, we demonstrated that the distribution width of T2 relaxation time (T2 heterogeneity) in the hippocampus predicted cognitive decline over a year in a group of people with MCI (Wearn et al., 2020a). We propose that T2 distribution widens because of different pathological hallmarks having opposing effects on T2, causing increased apparent heterogeneity without any change in absolute T2 (distribution midpoint). For example, non-haem iron, oligomers and plaques of β-amyloid (Aβ), and neurofibrillary tangles (NFTs) which build up around the MTL in early Alzheimer's disease (Braak and Braak, 1991, 1995; Selkoe and Hardy, 2016; Smith et al., 2010) all cause T2 to decrease (Meadowcroft et al., 2015; House et al., 2008). In contrast, tissue alterations preceding necrosis cause cell membrane breakdown and oedema which increase the motility of water within a given region, subsequently causing T2 to increase (Laakso et al., 1996; Symms et al., 2004). Gliosis, another hallmark of brain injury that commonly follows neurodegeneration is also indicated by increased T2 relaxation time (Briellmann et al., 2002; Ingelsson et al., 2004; Lee et al., 2013). These opposing factors necessitate examination of the heterogeneity of T2, rather than midpoint, for more accurate identification of microstructural impairment. This, we propose, is a reason for the lack of clear consensus from previous studies of T2 in AD, which exclusively look at absolute T2 (see Tang et al., 2018 for a review).

Our previous research focused on T2 changes in the hippocampus as a whole (Wearn et al., 2020a). However, the hippocampus is not a uniform structure, rather, it comprises cytoarchitectonic subfields with distinct cellular structure, connectivity, functionality, and disease susceptibility (Duvernoy et al., 2013). NFTs first build up in the transentorhinal region of the MTL (Braak and Braak, 1995), which roughly corresponds to Brodmann area 35 (BA35), but also includes some of the lateral portion of entorhinal cortex (EC) (Xie et al., 2018). They then spread through EC, then to CA1, subiculum, other CA regions and finally dentate gyrus (DG) (Braak and Braak, 1995, 1991; Fukutani et al., 1995, 2000). Many of these changes occur even before symptom onset (Braak and Braak, 1991; Jack et al., 2003; Fukutani et al., 1995), highlighting their potential as prodromal markers. Reflective of histopathology, hippocampal volume loss due to AD is widespread across the hippocampus but is generally non-uniform across subfields, with most atrophy seen in CA1 (Adler et al., 2018; La Joie et al., 2013; Mueller et al., 2010; Wolk et al., 2017; Kerchner et al., 2012; Sarazin et al., 2010; Frisoni et al., 2008, 2006). Volume loss is less severe in the hippocampus of people with MCI, often restricted to CA1 and, in many cases, the subiculum (Pluta et al., 2012; Mueller et al., 2010; Tang et al., 2014), with some evidence for a relative sparing of the stratum radiatum/lacunosum/moleculare (Su et al., 2018). This atrophy pattern has even been shown in people who subjectively report cognitive decline but who have normal cognition as measured by standard cognitive tests (Perrotin et al., 2015). For a review see de Flores et al. (2015b).

Literature on quantitative T2 in MTL subfields in the context of AD is limited to very few *ex vivo* studies (Huesgen et al., 1993; Antharam et al., 2012). As with the rest of the literature, these papers are focused on absolute T2. Antharam et al. (2012) do note that the distribution width of T2 within the main hippocampal subfields (CA4-DG and CA1–3) is wider in slices from AD patients than age-matched controls. However, they do not perform detailed analyses of the differences of distribution width between subfields. In a publication of pilot data from our group, Knight et al. (2019) concluded that T2 heterogeneity in MTL subfields can improve accuracy in distinguishing between healthy controls, those with MCI and Alzheimer's disease patients. We know of no other studies that have explored quantitative T2 in subfields of the MTL *in vivo*.

The analyses in this paper are presented in two parts. In the first part, we test whether differences in T2 heterogeneity between subfields could distinguish healthy ageing from MCI – important when considering T2 as a clinic tool to guide prognosis in MCI. We also analyse absolute T2 (distribution midpoint) to verify that it is heterogeneity and not absolute values that shift. Hypotheses:

1 The effect size of T2 heterogeneity increase in MCI (compared to healthy controls) will differ by subfield, reflective of the spatio-temporal progression of neurodegeneration in AD. Accordingly, we expect to see greatest T2 heterogeneity in MTL cortical regions (BA35 and EC), followed by CA and SUB regions, and the least amount of heterogeneity in DG.
2 T2 heterogeneity in MTL subfields will better predict cognitive decline than in whole hippocampus in people with mild cognitive impairment.

In the second part, we use path analysis to examine the likely temporal sequence of neuroanatomical and behavioural changes in ageing. These are important to understand so that we track the right process at the right disease stage. Hypotheses:

1 Greater T2 heterogeneity indicates early damage that will lead to macroscopic structural change, and therefore will statistically mediate the relationship between age and volume of MTL subfields.
2 Subfield volume is indicative of macroscopic structural change, and therefore will mediate the relationship between T2 heterogeneity and cognitive performance.

## 2 Material and methods

The following methods are adapted from those presented by Wearn et al. (2020a).

The analyses in this paper combine data from two prospective longitudinal studies similar in cohort demographics and study design. No participants took part in both studies. Both studies are detailed in the following section. Where data collected are not identical between cohorts, we have normalised equivalent metrics within cohort and combined data after normalisation.

### 2.1 Participants

Participants fulfilling the Petersen criteria (Albert et al., 2011) for diagnosis of MCI were recruited to both studies (Study 1: *n* = 30, Study 2: *n* = 29). Healthy older people (HC), with no history of memory problems or significant neurological disorders were recruited as controls to each study (Study 1: *n* = 61; Study 2: *n* = 56). All healthy controls had Montreal Cognitive Assessment (MoCA) ≥ 26 (study 1) or Addenbrookes Cognitive Examination 3 (ACE-III) ≥ 88 (study 2). 7 participants originally recruited as healthy controls in study 1 were found to have MoCA scores of < 26, so were reclassified as MCI (given the high sensitivity and specificity of the MoCA for detecting MCI at this threshold; 90% and 100%, respectively (Nasreddine et al., 2005)).

Subjects for both studies were recruited from local GP surgeries and memory clinics in the Bristol area (having received MCI diagnoses or reported memory problems), Join Dementia Research, Avon and Wiltshire Mental Health Partnership's Everyone Included system, an in-house database of volunteers, replies to poster adverts or through word of mouth. All patients provided informed written consent prior to testing as according to the Declaration of Helsinki. Ethical approval was given by Frenchay NHS Research Ethics Committee.

The current analyses included all participants who had both volumetry and T2 relaxometry data for hippocampal subfields, study 1 *n* = 91 (50 HC, 30 MCI), study 2 *n* = 66 (49 HC, 19 MCI). See Supplementary Tables 1 and 2 for demographic details of each study. A total of 20 MCI participants were followed-up after one-year (10 from each study), representing a relatively high dropout rate from the MCI cohort.

### 2.2 Cognitive testing

Cognitive function was tested at baseline and follow-up using the MoCA in study 1 and the ACE-III in study 2.

Participants in both studies carried out the paired associates learning (PAL) task of the CANTAB toolbox which has shown high sensitivity to cognitive impairment and daily functioning in dementia (Égerházi et al., 2007).

### 2.3 Imaging parameters

Scans for both studies were acquired on a Siemens Magnetom Skyra 3T system equipped with a parallel transmit body coil and a 32-channel head receiver array coil. The two studies used similar, but slightly different scanning protocols.

#### 2.3.1 Study 1

This protocol has been previously described by Knight et al. (2019). The imaging protocol included a 3D T1-weighted whole-brain magnetisation prepared rapid acquisition gradient-echo (MPRAGE) and 2D multi-contrast multi-spin-echo (CPMG).

MPRAGE: Coronal, whole-brain, repetition time (TR) 2200 ms, Echo Time (TE) 2.42 ms, Inversion time (TI) 900 ms, flip angle 9°, acquired resolution 0.68 × 0.68 × 1.60 mm, acquired matrix size 152 × 320 × 144, reconstructed resolution 0.34 × 0.34 × 1.60 mm (after two-fold interpolation in-plane by zero-filling in k-space), reconstructed matrix size 540 × 640 × 144, GRAPPA factor 2. Acquisition time: 5:25 min.

CPMG: Coronal, TR 4500 ms, TE 12 ms, number of echoes 10, echo spacing 12 ms, acquired resolution 0.68 × 0.68 × 1.7 mm inclusive of 15% slice gap, acquired matrix size 152 × 320, 34 slices, interleaved slice order, reconstructed resolution 0.34 × 0.34 × 1.7 mm (after two-fold interpolation in-plane by zero-filling in *k*-space, and inclusive of 15% slice gap), reconstructed matrix size 540 × 640, 34 slices, GRAPPA factor 2. Acquisition time: 11:07 min.

#### 2.3.2 Study 2

This protocol has been previously described by Wearn et al. (2020a).

The imaging protocol included a 3D T1-weighted whole-brain MPRAGE and 2D multi-contrast turbo spin-echo (TSE).

MPRAGE: Sagittal, whole-brain, TR 2200 ms, TE 2.28 ms, TI 900 ms, flip angle 9°, FOV 220 × 220 × 179 mm, acquired resolution 0.86 × 0.86 × 0.86 mm, acquired matrix size 256 × 256 × 208. Acquisition time: 5:07 min.

Multi-contrast TSE: Coronal, TR 7500 ms, number of echoes: 3, TEs of 9.1, 72 & 136 ms, acquired resolution 0.69 × 0.69 × 1.5 mm, reconstructed resolution 0.34 × 0.34 × 1.5 mm (after 2-fold interpolation in-plane by zero-filling in *k*-space, and inclusive of 15% slice gap), GRAPPA factor 2, FOV 220 × 220 × 34, acquired matrix size 270 × 320 × 58. Acquisition time: 5:09 min.

CPMG and TSE scans were not ‘whole-brain’, their coverage only extending approx. 1 cm beyond anterior and posterior ends of the hippocampus. These scans were tilted such that the hippocampal body lay perpendicular to the slice acquisition plane.

The two distinct methods of measuring quantitative T2 (CPMG vs TSE) will give inherently different values for T2 midpoint and heterogeneity between studies (See supplementary information). Relationships to variables such as age and cognitive score should be similar, given they are sensitive to the same tissue properties.

### 2.4 Imaging analyses

All analyses were performed at CRICBristol in a Linux cluster environment. All analyses were carried out in single-subject native space.

CPMG and TSE scans were brain-extracted using FSL's *bet2* on the first echo in the series (Smith, 2002). All extracted images were visually inspected for quality and rerun with different fractional intensity thresholds or gradient parameters where necessary. Fractional intensity threshold was typically set between 0.2 and 0.3. MPRAGE images were brain-extracted using *vbm8bet* (in-house script) and bias-field-corrected using FSL FAST (Zhang et al., 2001). T2 maps were created in MATLAB from multi-echo sequences by fitting logarithmic-space mono-exponential decay functions to each voxel series (overall summary of T2 calculation is shown in Knight et al. (2019)). The first echo of CPMG was always excluded. A sum-of-echoes image was created in order to have one structural image representing the entire multi-echo sequence. This image was used for segmentation.

**Fig. 1:**
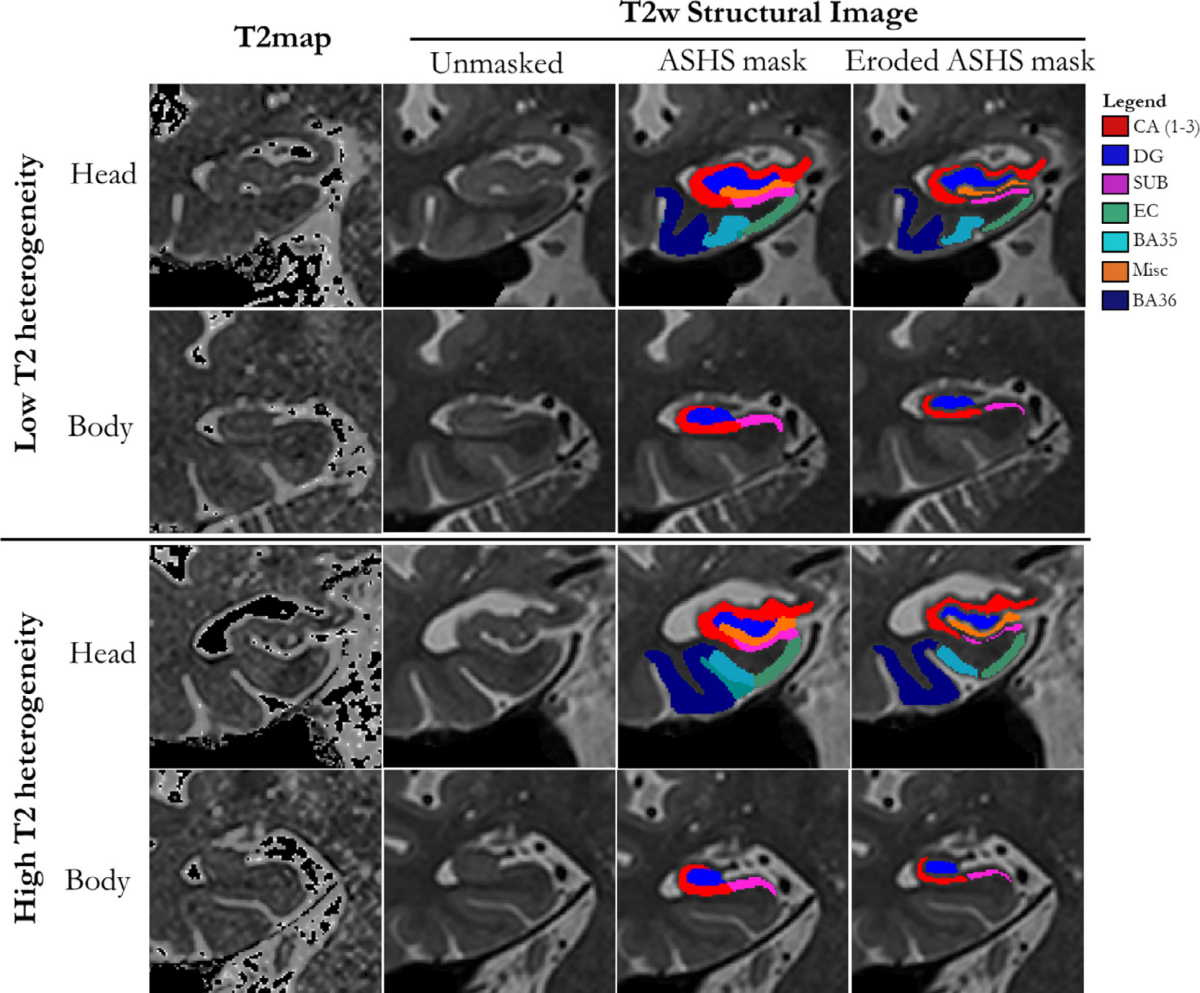
Coronal slices of MTL in two example participants with high and low T2 heterogeneity. Each row shows (left-right) T2 maps, unmasked T2-weighted structural images (summed-over-echoes), ASHS masks, and masks eroded to reduce influence of partial voluming. Top two rows show images from an individual with low T2 heterogeneity (Healthy control, age 66). Bottom two rows show images from an individual with high T2 heterogeneity (MCI, age 84). Slices from hippocampal head and body are shown for each participant. In ‘High T2 heterogeneity’ T2 maps, a higher prevalence of bright and dark spots are visible, in comparison to the ‘Low T2 heterogeneity’ T2 maps. DG = Dentate Gyrus, CA = Cornu Ammonis 1–3, SUB=Subiculum, EC = Entorhinal Cortex, BA = Brodmann Area. Misc and BA36 were excluded from all analyses. [COLOUR FIGURE].

The hippocampus was automatically masked using the Automatic Segmentation of Hippocampal Subfields (ASHS) software package (Yushkevich et al., 2015) (version: rev103, dated 12/06/2014; UPENN memory centre atlas dated 16/04/2014). This atlas contains a demographically similar template set to the present studies (healthy older adults and people with MCI). ASHS has demonstrated high accuracy whilst minimising subjective rater bias, without the need for group blinding (Example output shown in Fig. 1). CA1, CA2 and CA3 were pooled to create a total “CA” mask, given the small size of CA2 and CA3. The outermost 1 voxel layer of each subfield was eroded before T2 histograms were calculated, in order to minimise the effects of partial voluming and extraneous brain areas confounding T2 histogram analyses (shown in Fig. 1). Pilot data (Knight et al., 2019) show that using this method, extreme T2 values are not located around the outside border of the subfields, which would indicate strong partial voluming effects. Eroded subfield masks were overlaid onto T2 maps, giving a value of T2 for each voxel within each subfield. Depending on subfield shape, 20–50% of voxels are removed using this method, leaving sufficient numbers from which to estimate distribution statistics.

### 2.5 Modelling T2 heterogeneity

T2 distribution histograms were modelled as loglogistic distributions within each subfield, as this was found to be the best fitting overall model in the whole hippocampus (Wearn et al., 2020a). Log-logistic distribution is defined as:

$$f\;\left(x\mu ,\sigma \right)=\;\frac{1}{\sigma }\frac{1}{x}\frac{\text{exp}\left(z\right)}{{\left[1+\text{exp}\left(z\right)\right]}^{2}},\;where\;z=\;\frac{\log\left(x\right)-\;\mu }{\sigma }$$

where μ and σ denote the log-median value (midpoint) and distribution shape (heterogeneity), respectively. These descriptors of log-logistic distribution are analogous to median and median absolute deviation on a normal distribution. The models are described in more detail by Wearn et al. (2020a).

### 2.6 Statistical analysis

Volumes, T2 parameters and general cognitive scores (MoCA / ACE-III) were pooled between studies after being converted into Z-scores for each study separately, with the left DG of healthy controls of each study as a reference point. In doing this, group, subfield, and hemisphere differences were maintained within each population.

We used a mixed model analysis, using the ‘*fitlme’* MATLAB function, to assess differences in T2 parameters between groups (HC, MCI), subfields (DG, CA, SUB, EC, BA35), hemispheres (left, right) and the effect of age. Full factorial models were created (assessing intercept and all possible interactions of aforementioned variables) with random effects of subject, subject*subfield interaction, and subject*hemisphere interaction. Each random effect significantly improved the model fit (as measured by AIC), without overparameterizing the model (confirmed by checking the hessian matrix and AIC). Age of the entire cohort was converted to a *Z*-score before being entered into the model. Models were created using Restricted Maximum Likelihood Estimation. Degrees of freedom were calculated using Satterthwaite approximation. A separate model was created for each of T2 midpoint (T2μ) and T2 heterogeneity (T2σ). Because of the inclusion of age in the models, all results are appropriately corrected for the effect of age.

Between-group *post hoc* comparisons were assessed using independent-samples *t*-tests. Cohen's *d* values for each comparison are reported as a measure of effect size. *Post hoc* effects of age in each subfield are assessed using linear regression.

Ability of volume and T2 in MTL subfields to predict cognitive decline (as measured by MoCA / ACE-III) was assessed using stepwise linear regression with forward selection. Forward stepwise regression works by first adding the variable (subfield) which best improves model fit (if any), then continuing to add variables which further significantly improve model fit. The criteria for adding more variables was a significant F statistic at the *p* < .05 level. We entered follow-up cognition as the dependant variable and baseline cognition and age as fixed covariates:

$$\begin{array}{ccc} & & \text{Follow}\;\text{-}\;\text{up}\;\text{Cognition}=\beta_{intercept}+\;\beta_{age}\left(\text{Age}\right)\\ & & \;+\;\beta_{BLCog}\left(\text{BaselineCognition}\right)+[\beta_{CA}\left(\text{CA}\right)+\beta_{DG}\left(\text{DG}\right)\\ & & \;+\beta_{SUB}\left(\text{SUB}\right)+\beta_{EC}\left(EC\right)+\beta_{BA35}\left(BA35\right)+\;\beta_{Hipp}\left(Hipp\right)]+\text{error} \end{array}$$

Parameters in square brackets represent those entered in a stepwise fashion. ‘Hipp’ represents total hippocampus. This analysis was performed for the MCI cohort only given the greater variability within this group. Z-scores for this analysis were calculated relative to each study's MCI population only. This method of predicting follow-up cognition whilst correcting for baseline cognition represents a more precise and less biased way of assessing cognitive change over time, compared to using change scores, as described by Vickers and Altman (2001).

We assessed the relationship between age, subfield T2 heterogeneity, subfield volume (corrected for intracranial volume (ICV)) and memory using path analysis. Memory was assessed using two measures of performance on the PAL task – total accuracy and mean reaction time across all trials. We took a semi-supervised approach when defining the model paths. The direction of the arrows was predetermined by theory; T2 heterogeneity is caused by microstructural changes that precede significant atrophy. In this regard, volumes were not allowed to predict T2. Memory scores were not allowed to predict structural variables, and, for obvious reasons, no variable was allowed to predict age. All direct effects in this direction were modelled and compared. Initially the model was run with both PAL scores in a single model, feeding into a single latent variable representing overall PAL score. However, we observed poor factor loadings of each score onto the latent variable (<0.60), so ran two separate models instead each with a single PAL outcome measure. Error terms were added to all variables except age and covariances and modification indices were calculated between all terms. All error term pairs within a structural measure (T2σ or volume) that had significant modification indices were allowed to covary, substantially improving overall model fit. In other words, subfields were allowed to covary within (but never between) each MRI modality. We observed poor model fit when data was entered as *Z-*scores normalised to left DG of HCs of each study as described above. We therefore normalised each structural measure to only the respective HC population, such that the mean ± standard deviation for each structural measure within any given subfield was 0 ± 1. In other words, differences between subfields were not considered by the model. This improved model fit to acceptable levels and should be considered in interpretation of the data.

We ran partial correlations to explore the relationship between absolute volume (uncorrected for intracranial volume) and T2 heterogeneity, correcting for age in healthy controls. The results of this analysis are presented in supplementary Table 8. Finally, we compared SNR between HC and MCI groups, the results of which are presented in Section 5 of supplementary material.

All reported p-values are two-tailed. Where possible we have used comprehensive models, to minimise the need for multiple comparison corrections. False discovery rate (FDR) correction (Benjamini and Hochberg, 1995) was applied to individual paths and indirect paths in the path analysis, in line with guidance from Cribbie (2007). Data handling and storage was carried out using MathWorks MATLAB 2015a (with statistics and machine learning toolbox) and Microsoft Excel 2016. Mixed models were created and assessed in MathWorks MATLAB 2018a. Other statistical analyses were performed in IBM SPSS Statistics 24. Graphs were produced using GraphPad Prism v8.

## 3 Results

### 3.1 Participant demographics

Demographic information for the cohort is displayed in Table 1. Our healthy control and MCI groups are closely matched for age and years of education. Details of cohorts in study 1 and study 2 are separately displayed in supplementary Tables 1 and 2.

**Table 1:** Participants demographics Cognitive score was calculated using MoCA (study 1) and ACE-III (study 2). Because of the different measures used between studies, each score was first normalised to each study's respective HC group, before being pooled here. Asterisks represent unpaired *t*-tests between groups (*p < .05, ***p < .0001). YOE = Years of Education, HC = Healthy Control, MCI = Mild Cognitive Impairment.

	HC	MCI	Total
*N* (male: female)	99 (47:52)	49 (27:22)	148 (74:74)
Age (years)	69.2 ± 8.55	72.2 ± 9.03	70.2 ± 8.79
YOE	15.8 ± 3.13	*14.2 ± 2.81	15.3 ± 3.11
Cognitive score (normalised to HC)	.000 ± 1.00	***−3.75 ± 2.42	−1.25 ± 2.39

### 3.2 Part 1: T2 changes in MCI

#### 3.2.1 T2 heterogeneity

**Fig. 2:**
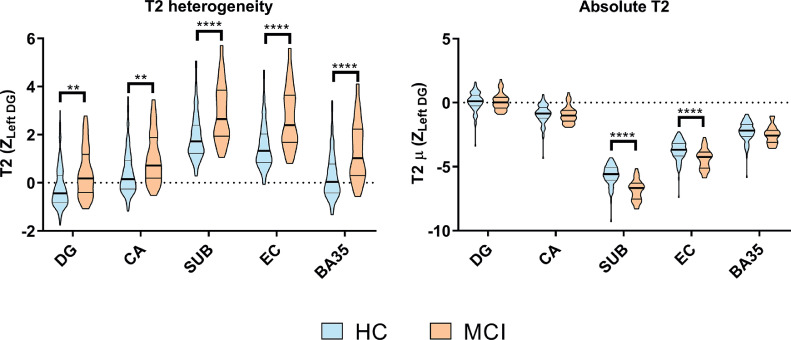
Subfield comparisons for T2 heterogeneity and absolute T2. Violin plots showing group & subfield differences (pooled across hemispheres & corrected for age) for absolute T2 and heterogeneity marginal means. HC = healthy older control; MCI = mild cognitive impairment. Stars represent *p*-values from post hoc two-way ANOVA tests to compare subfield groupwise differences. **p < .01; ***p < .001; ****p < .0001. [COLOUR FIGURE].

In addition to the overall effects of group (*F*(1, 144)=15.2, *p*=.0001) and subfield (*F*(4, 576)=238, *p* < .0001; explored further in supplementary information) and in line with hypothesis 1, the mixed model analysis revealed a significant group*subfield interaction (*F*(4, 576)=2.92, *p*=.020). This effect did not vary according to hemisphere so a two-way ANOVA on pooled hemispheres was conducted with predicted values from the mixed model analysis, with pairwise group comparisons for each subfield (Fig. 2). This test revealed a significantly greater T2 heterogeneity in the MCI group in all subfields but with varying effect sizes (DG: *t* = 3.46, p_corr_ =0.004, Cohen's *d* = 0.62; CA: *t* = 3.55, p_corr_ =0.003, Cohen's *d* = 0.63; SUB: *t* = 5.07, p_corr_ <0.0001, Cohen's *d* = 0.92; EC: *t* = 5.88, p_corr_ <0.0001, Cohen's *d* = 1.07; BA35: *t* = 5.22, p_corr_ <0.0001, Cohen's *d* = 0.95, all adjusted using Bonferroni correction for multiple comparisons).

We also found a significant main effect of age (*F*(1, 144)=33.2, *p* < .0001) and an interaction between subfield and age (*F*(4, 576)=5.16, *p*=.0004). Older people had significantly increased T2 heterogeneity in all subfields. In increasing order of strength of association, the associations were as follows: SUB (*R^2^*=0.146, *F*(1, 146)=25.0, p_corr_<0.0001), DG (*R^2^*=0.159, *F*(1, 146)=27.6, p_corr_<0.0001), CA (*R^2^*=0.163, F(1, 146)=28.5, p_corr_<0.0001), EC (*R^2^*=0.168, *F*(1, 146)=29.6, p_corr_<0.0001) and BA35 (*R^2^*=0.206, *F*(1, 146)=37.9, p_corr_<0.0001).

Finally, we observed no interactions between group and age (*F*(1, 144)=1.27, *p*=.261) or group and hemisphere (*F*(1, 144)=0.135, *p*=.714) nor were there any three- or four-way interactions for T2 heterogeneity.

#### 3.2.2 Absolute T2

For comparison, we also explored subfield-specific changes in absolute T2. We observed an overall difference between HC and MCI groups on absolute T2 (*F*(1, 144)=5.33, *p*=.022), and a substantial overall difference in absolute T2 between subfields (*F*(4, 576)=1220, *p* < .0001; explored further in supplementary information). We found a group*subfield interaction (*F*(4, 576)=9.21, *p* < .0001) driven by a specific low T2μ in the subiculum and EC of the MCI group (SUB: *t* = 8.45, p_corr_<0.0001, Cohen's *d* = 1.47; EC: *t* = 4.73, p_corr_<0.0001, Cohen's *d* = 0.82). In no other subfield was there any difference between groups.

We observed a significant main effect of age on absolute T2 (*F*(1, 144)=4.86, *p*=.029), and a significant interaction between subfield and age (*F*(4, 576)=7.23, *p* < .0001). However, in no subfield was there any statistically significant association between age and absolute T2 (all subfields: *p* > .080).

We observed no interactions between group and age (*F*(1, 144)=0.029, *p*=.865) or group and hemisphere (*F*(1, 144)=2.99, *p*=.086) nor were there any three- or four-way interactions for absolute T2.

### 3.3 Predicting cognitive change over time

In order to identify whether MTL subfields could predict cognitive decline, we ran three stepwise linear regressions to predict cognitive score of people with MCI after one year. One model was run for each MRI modality: volume, T2 heterogeneity, absolute T2, whilst always accounting for baseline cognitive score and age. Age and baseline cognition alone were unable to significantly predict follow-up cognitive score (*R^2^*=0.092, *F*(2, 19)=0.859, *p*=.441; AIC=29.9). In other words, age alone was unable to predict the degree of cognitive change over the year.

**Fig. 3:**
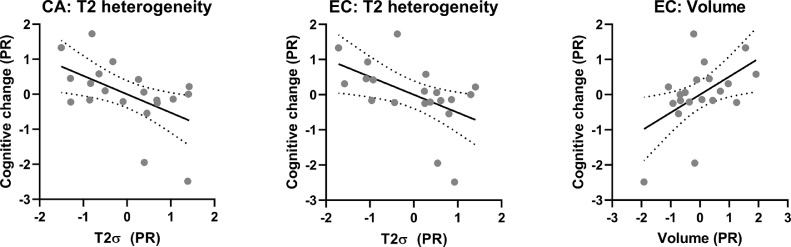
Subfield structure predicting cognitive decline in people with MCI. Partial residual (PR) plots of significant predictors from stepwise linear regression models. Graph shows linear regression lines (solid lines) ± 95% confidence intervals (dotted lines). Significant predictors were only created for T2 heterogeneity and Volume models. Volumes were corrected for intracranial volume. [COLOUR FIGURE].

Of all three modalities, only T2 heterogeneity and volume showed any ability to predict cognitive change over time. Greater T2 heterogeneity in CA was as a significant predictor of cognitive decline (β_CA_=−0.605, *p*=.025) (Fig. 3). However, the overall model was not statistically significant (*R^2^*=0.344, *F*(3, 19)=2.79, *p*=.074, AIC=25.4), indicating relatively weak predictive power. T2 heterogeneity in entorhinal cortex possessed similar predictive power to CA (β_EC_=−0.567, *p*=.031) (Fig. 3), but was not added to the model as it did not describe enough *additional* variance on top of that described by CA. No additional subfields sufficiently increased the model fit to qualify for entry into the model, or were significant alternative predictors.

Similarly, EC volume was entered as sole predictor of greater cognitive decline in the volume model (β_EC_=0.595, *p*=.030). However the full model was not statistically significant (*R^2^*=0.329, *F*(3,19)=2.62, *p*=.087, AIC=25.8), indicating a weak association between subfield volume and cognitive change. Absolute T2 was unable to predict cognitive decline over the year, with no subfields being entered into this model.

### 3.4 Part 2: sequential relationships across brain structure and behaviour

We used path analysis to explore predicted relationships between age, T2 heterogeneity, subfield volume (relative to intracranial volume) and cognition. We took a semi-supervised approach when defining the model paths. The direction of the arrows was predetermined on the theoretical basis that T2 heterogeneity is likely caused by microstructural changes that precede significant atrophy. Memory scores were outcome variables, so were not allowed to predict structural variables, and no variable was allowed to predict age. All direct effects in this direction were modelled and compared. Two separate models were created, one for each of the PAL output measures (See Methods). After applying covariance structures to error terms between subfields, model fit was good with various fit parameters falling within an acceptable range (*χ^2^*=30.9, df = 20, *p*=.056; C_min_/DF=1.55; GFI=0.951, AGFI=0.811; CFI=0.986; RMSEA=0.076 ± 90% CI [<0.001–0.126]; p_close_=0.199). Model fit was identical between the two models. Covariance matrices can be found in supplementary information (Supplementary Tables 4 & 5).

**Fig. 4:**
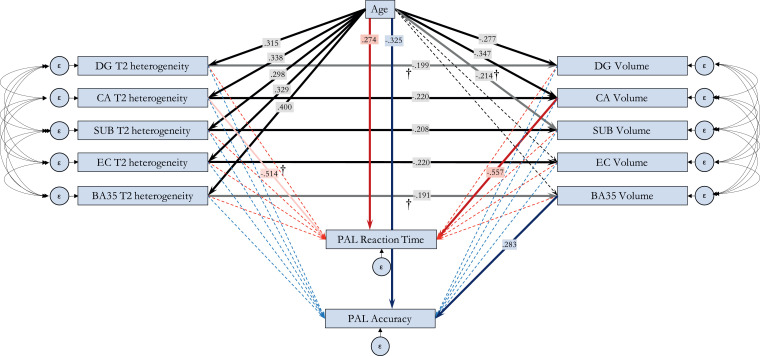
Path analysis showing the relationship between Age, T2 heterogeneity, volume and memory in MTL subfields in healthy older controls. Bold arrows represent statistically significant relationships (*p* < .05), with standardised *B* values indicated in overlaid boxes. Paths that do not survive FDR correction for multiple comparisons are faded and marked with †. Two models were run, each assessing one outcome measure of the PAL task. Black lines represent paths shared between the models. Unique paths to each model are shown in red (PAL reaction time as dependant variable) and blue (PAL Total Accuracy as dependant variable). Curves lines represent error term covariances defined in the model. All subfield volumes were normalised to ICV prior to entering into the model. Full statistics are shown in Supplementary Table 3. [COLOUR FIGURE] (For interpretation of the references to color in this figure legend, the reader is referred to the web version of this article.).

The final path analysis models (Fig. 4) reveal the following relationships, for which statistics are shown in Table 2 and Supplementary Table 3. Age is a significant positive predictor of T2 heterogeneity in all MTL subfields. In turn, T2 heterogeneity within each subfield is a significant negative predictor of subfield volume, although for DG and BA35, this effect is weaker and does not survive correction for multiple comparisons. The only significant direct effects of age on subfield volume are seen with DG and CA (where greater age predicts smaller volumes), however, significant indirect effects are seen between age and subfield volume within all subfields (Table 2). T2 heterogeneity therefore strongly, albeit partially, mediates the relationship between age and volume.

**Table 2:** Summary statistics of key indirect paths. All paths remain statistically significant after FDR correction for multiple comparisons (corrected *p*-value threshold = 0.011).

Indirect path		Standardised estimate	*P*-value
Age → T2 heterogeneity → Volume			

	DG	−0.063	*.005
	CA	−0.074	*.003
	SUB	−0.062	*.008
	EC	−0.072	*.008
	BA35	−0.076	†.022

T2 heterogeneity → Volume → PAL			

PAL Accuracy	DG	.046	†.020
	CA	−0.042	.123
	SUB	−0.029	.129
	EC	.013	.576
	BA35	−0.054	†.025
PAL Reaction Time	DG	−0.038	.141
	CA	.123	*.003
	SUB	−0.033	.169
	EC	−0.011	.486
	BA35	.018	.237

Age → T2 heterogeneity → Volume → PAL			

PAL Accuracy	DG	.015	†.014
	CA	−0.014	.100
	SUB	−0.009	.094
	EC	.004	.539
	BA35	−0.022	†.018
	Total path (all subfields)	−0.005	.153
PAL Reaction Time	DG	−0.012	.110
	CA	.041	*.003
	SUB	−0.010	.121
	EC	−0.004	.435
	BA35	.007	.199
	Total path (all subfields)	.005	.174

No significant direct effects that survived multiple comparisons were observed between T2 heterogeneity and either PAL score (Fig. 4). However, a direct negative effect of CA volume on PAL mean reaction time was observed, as well as a direct positive effect of BA35 volume on PAL accuracy. The indirect relationship between T2 heterogeneity in CA and PAL reaction time via volume is statistically significant, indicating a role of volume as a mediator between T2 heterogeneity and cognition. However, the same pathway for BA35 does not survive correction for multiple comparisons (Table 2). Variation in BA35 volume therefore seems to predict PAL accuracy independently of T2 heterogeneity and age.

Finally, outside of MTL subfield structure and microstructure, we also observe direct effects of age on PAL scores, with greater age predicting poorer scores on both measures (Table 2).

This all serves to show a pathway of the effect of age on cognition, first through its increasing of T2 heterogeneity in subfields, which in turn reduces the volume of each subfield, which then, in a subfield-specific manner, reduces cognitive functionality. The full indirect pathway from age, through T2 heterogeneity, to volume and finally to PAL score is statistically significant for the pathway through CA for predicting PAL reaction time (Table 2), however the pathway through DG and BA35 for predicting PAL accuracy does not survive correction for multiple comparisons. Outside any MTL subfield paths, there remains a direct effect of age on PAL score, whereby greater age predicts poorer performance (lower accuracy, longer reaction times), suggesting that age affects PAL performance through non-hippocampal as well as hippocampal pathways.

We ran the models again on the MCI group data to explore any differences in path structure to the HC models. The main changes in this model were the lack of a relationship between T2 heterogeneity and volume in most subfields (SUB, EC, BA35), and the loss of any relationship between PAL scores and both age and subfield structure measures. We also ran a model with the volumes allowed to predict T2 heterogeneity. This reverse path analysis shows that T2σ does not have the same mediatory effect on volume that volume has on T2σ and has poorer model fit parameters. Model path diagrams can be seen in supplementary information (supplementary Figs. 1 & 2 and supplementary Tables 4 & 5).

## 4 Discussion

Here we have shown that T2 heterogeneity in the MTL increases with cognitive impairment in a subfield-dependant manner in line with progression patterns of Alzheimer's pathology. T2 heterogeneity in CA1-3 and volume of entorhinal cortex showed some ability to predict cognitive decline, where absolute T2 could not, however further studies are required to verify this result. Furthermore, we describe a mechanism by which cognitive ability deteriorates with age through direct effects on T2 heterogeneity, which lead to the changes in volume which in turn lead to cognitive decline. These findings are discussed in detail below.

### 4.1 Subfield-specific differences in T2 heterogeneity in MCI and with age

T2 heterogeneity was greater in MCI across all subfields of the MTL, however the magnitude of this effect differed between subfields. The smallest increase was seen in DG, and the largest in EC and BA35. This is in line with literature on the timing of deposition of NFTs throughout the course of AD (Braak and Braak, 1991, 1995), whereby the transentorhinal region (comprising BA35 and lateral EC) is affected first, and DG pathology is only detectible later on. DG is also one of the last MTL regions to exhibit volume loss (Daugherty et al., 2015). This accordance between the pattern of T2 heterogeneity differences and AD neuropathological progression supports T2 heterogeneity as a means to detect pathologically relevant change before the onset of dementia.

This is in contrast to absolute T2, which is only different between healthy controls and people with MCI in subiculum and entorhinal cortex (where it is decreased in MCI). The effect may be explained by an increase in iron and compact amyloid plaques, which have been seen to be increased in SUB and CA1 regions compared to DG and CA3 in a mouse model of AD (Reilly et al., 2003). Even though we cannot directly show the prevalence of AD pathology in our MCI sample, these results are in line with this group being at higher risk for displaying AD pathology.

Our findings support our proposal (Knight et al., 2019; Wearn et al., 2020a) that T2 heterogeneity defines hippocampal integrity at the subfield level better than absolute T2. In further support of this model, two studies of absolute T2 in hippocampal subfields *in vitro* (Huesgen et al., 1993; Antharam et al., 2012) note a lack of any consistent pattern between healthy control and Alzheimer's slices in any subfield.

### 4.2 Clinical utility of T2 heterogeneity in MTL subfields

T2 heterogeneity in CA and EC and volume of EC showed some ability to predict cognitive decline, where absolute T2 could not, however further studies are required to verify this result as the overall models were not statistically significant. Measuring T2 heterogeneity in CA or EC may provide a novel way of identifying those who have MCI due to incipient Alzheimer's disease as opposed to other causes, though further testing is required to confirm to what degree this measure is specific to detecting Alzheimer's over other pathologies.

It is important to note that other subfields were unable to improve model fit as a likely result of shared variance being explained by each subfield. In other words, we cannot conclude that the predictive power provided by CA was ‘significantly greater’ than that provided by other subfields. Rather, other subfields did not provide a significant amount of additional information on top of that provided by heterogeneity in CA. Indeed, T2 heterogeneity in EC had similar, statistically significant predictive power, just behind that of CA.

EC structure has repeatedly been shown to better predict conversion to Alzheimer's disease than hippocampal structure (most often measured by volume or thickness, reviewed by Zhou et al. (2016)). However, it is interesting to note that EC performs better as a predictor than BA35, despite BA35 supposedly being an earlier NFT deposition site in AD. One reason could be that the transentorhinal cortex spans both EC and BA35 regions, and that subsequent microstructural changes are more widespread across EC compared to BA35 in MCI, supported by histological data (Braak and Del Tredici, 2015).

The use of T2 heterogeneity is highly translatable to clinical settings. MRI is standard practice in improving the accuracy of a diagnosis of AD. The MRI scan necessary to calculate quantitative T2 can be completed within a few additional minutes of a standard clinical MRI (a multi-echo T2 sequence of sufficient resolution is all that is required). The same high-resolution scan can be used to automatically segment subfields of the MTL e.g. using ASHS (Yushkevich et al., 2015). Although the use of MRI in people with dementia can sometimes be tricky due exacerbated feelings of claustrophobia and confusion, this MRI protocol would be most useful in prodromal stages of the disease, before significant MTL volume loss and symptom severity, minimizing these complications. A cheaper theoretical screening test with high sensitivity for detecting pathology such as has been shown with tests of accelerated forgetting on word list tasks (Wearn et al., 2020b; Weston et al., 2018) or a blood test (Toombs and Zetterberg, 2020) may identify individuals who should qualify for an MRI scan sensitive to very early pathological hallmarks.

Our method of measuring heterogeneity of an MRI signal is arguably a form of texture analysis, a technique for detecting microstructural changes on MRI whose clinical applications are increasingly a topic of interest (for a review see Cai et al. (2020)). Zhao et al. (2020) highlight the use of ‘radiomic biomarkers’ (a method of texture analysis) as a robust method of predicting longitudinal change, and genetic risk for Alzheimer's disease. Similarly, Sørensen et al. (2017) find that various measures of hippocampal structure including volume and texture can accurately discriminate healthy older people from those with MCI and Alzheimer's disease. To our knowledge, no study has attempted texture analysis using quantitative T2, or in MTL subfields, making this study the first of its kind. This appears to be an emerging field with increasing potential for scientific interest and clinical application.

Although we have focussed on T2 changes due to Alzheimer's pathology, T2 heterogeneity is a novel measure which could be applied to any neurological disease characterised by microstructural changes, including other dementias, acute stroke (as demonstrated by Norton et al. (2017)), epilepsy or schizophrenia. Future research could utilise T2 heterogeneity to easily probe microstructural abnormalities. This study builds on our past analyses of T2 heterogeneity by highlighting how it can reveal structural changes on an even finer scale, indicating its usefulness in disorders where brain damage is highly localised.

### 4.3 Subfield-specific contributions to memory

We aimed to better understand the relationship between T2 heterogeneity, volume, age and cognitive ability in cognitively healthy older people. To summarise, our path analysis revealed the following pattern of relationships. T2 heterogeneity mediates the negative relationship between age and volume. Volume in turn mediates the negative relationship between T2 heterogeneity and memory (PAL reaction time), in a subfield-specific manner. In line with hypotheses 3 and 4, increased T2 heterogeneity therefore seems to represent a state of structural damage which may give rise to the volumetric changes which have the most profound impact on memory and cognition. Interestingly, we do still find a direct association between age and volume in CA and DG, indicating only partial mediation by T2 heterogeneity in these regions. This suggests that there are other age-mediated volumetric changes which are not accurately reflected by changes in T2 heterogeneity.

Our results reveal potential subfield-specific associations with memory scores unlike previous similar models that have looked at whole hippocampus only (Rodrigue et al., 2012). Longer reaction times were associated with subtle damage to (higher T2 heterogeneity in) the CA region, whereas poor accuracy was associated with low BA35 integrity. These findings, although subject to confirmation in independent cohorts, indicate a point of interest for future studies to examine hippocampal subfield specificity to behavioural measures, and to further explore to what degree individual subfield structural changes may indicate early signs of pathology.

The same model assessed in our MCI population revealed a substantially different picture. Although the relationship between age and T2 heterogeneity was still very much present, T2 heterogeneity was not such a clear mediator of the age-volume relationship. Furthermore, no associations were observed with either cognitive measure. Our sample size in this group was much smaller, so we hesitate to draw firm conclusions from this analysis, however this may indicate a disjoint between hippocampal structure and cognitive performance in this group, indicating that other brain networks, such as those involving thalamus (Aggleton, 2014; Aggleton et al., 2016), become critical to maintain cognitive functioning in mild cognitive impairment.

### 4.4 Limitations

The main limitation is the lack of biomarker status availability for people with MCI in this study (assessed either from Positron Emission Tomography or CSF analysis). Therefore, we cannot be certain as to the exact proportion of those who have incipient dementia. We suspect that T2 heterogeneity in entorhinal cortex would identify those who do have incipient Alzheimer's disease, as EC is one of the earliest sites of pathological change. This is supported by the ability of EC T2 heterogeneity in predicting cognitive decline. However, co-pathologies are almost certainly present in the overall cohort which may include other dementias or undiagnosed microbleeds.

Additionally, both studies utilised different scanning sequences for assessing quantitative T2, giving inherently different absolute T2 values. For this reason, we were careful to normalise results from each cohort before combining them. Although a potential confounding factor, we have noted in our previous study that effects of T2 heterogeneity do not appear to be specific to a sequence (Wearn et al., 2020a). Rather, we provide evidence that the different sequences are sensitive to the same physiological changes. This supports the translatability of this measure to clinical settings, where available sequences for measuring quantitative T2 may vary between sites.

We also note a potential selection bias in our 1-year follow-up analysis, as we experienced a relatively high dropout rate of our MCI cohort. Although reasons for not returning were never formally quantified, this was occasionally due to the participant feeling unable to attend the second session due to significant cognitive and/or functional decline. Those who declined the most may therefore be simply missed out of the current analyses. We expect the result of this bias to minimise the ability of our MRI variables to predict decline, so it is testament to the clinical potential of T2 heterogeneity that it can still predict cognitive decline in this cohort.

We acknowledge that our masking procedure of hippocampal subfields (automated using ASHS) has only been verified for measuring shape and volume, not T2 heterogeneity. Measures could feasibly be biased due to the inclusion of extraneous brain regions or subject to error from surrounding regions through partial voluming. We have minimised the risk of these factors by eroding each subfield mask by its outermost voxel. Furthermore, we find no evidence of a relationship between absolute subfield volume and T2 heterogeneity, indicating that the two measures are distinct, and not confounded by one another (supplementary information Section 4).

Finally, with our present analyses we cannot make conclusions as to the specificity of these changes to hippocampal subfields, as opposed to other brain regions not implicated in early Alzheimer's disease. This was not possible in this study as the acquisition area of each of the multi-echo T2 scans did not span the whole brain, and so selection of an appropriate control region was limited. Future studies should aim to explore T2 dynamics in other brain regions.

## 5 Conclusions

The analyses presented in this paper comprise the first detailed exploration of quantitative T2 across subfields of the medial temporal lobe in older people with and without cognitive impairment. We support previous evidence that absolute T2 is not a sensitive marker of early pathology, rather, heterogeneity of T2 is much more sensitive to early pathological change. We demonstrate that T2 heterogeneity differs between subfields in a manner which reflects the order of NFT deposition in prodromal AD. We provide evidence that although T2 heterogeneity increases with age in all subfields, the degree to which this occurs is subfield-dependant and is strongest in MTL cortical regions (EC and BA35). In contrast, we do not see systematic evidence of a relationship between age and absolute T2. Using path analysis, we describe a pathway through which cognition is significantly affected by age through direct effects on T2 heterogeneity in cognitively healthy older people, which in turn has direct effects on volume which lead to changes in cognition, supporting the idea that T2 changes precede and lead to volumetric changes. Finally, we provide evidence that increased T2 heterogeneity in CA1-3 and entorhinal cortex may indicate future cognitive decline in people with MCI, whereas measures of absolute T2 are unable to predict such decline.

## Declarations

### Ethics approval and consent to participate

All patients provided informed written consent prior to testing. Ethical approval was given by Frenchay NHS Research Ethics Committee.

### Consent for publication

Not applicable

### Data and code availability

The datasets used during the current study are available from the corresponding author on reasonable request and setup of a formal data sharing agreement. Code used to compute T2 maps is available to download from https://data.bris.ac.uk/data/dataset/1bjytiabmtwqx2kodgbzkwso0k (Wearn et al., 2017).

## Funding

This research was funded in part by the Wellcome Trust [Grant number 109,067/Z/15/AI]. For the purpose of open access, the author has applied a CC BY public copyright licence to any Author Accepted Manuscript version arising from this submission. This study also was funded by Alzheimer's Research UK and BRACE.

## CRediT authorship contribution statement

**Alfie R. Wearn:** Conceptualization, Data curation, Formal analysis, Funding acquisition, Investigation, Methodology, Project administration, Resources, Software, Visualization, Writing - original draft, Writing - review & editing. **Volkan Nurdal:** Data curation, Investigation, Resources. **Esther Saunders-Jennings:** Data curation, Investigation, Resources. **Michael J. Knight:** Data curation, Resources, Software, Writing - review & editing. **Christopher R. Madan:** Formal analysis, Methodology, Writing - review & editing. **Sean-James Fallon:** Formal analysis, Methodology, Writing - review & editing. **Hanna K. Isotalus:** Investigation, Resources. **Risto A. Kauppinen:** Funding acquisition, Methodology, Supervision, Validation, Writing - review & editing. **Elizabeth J. Coulthard:** Conceptualization, Funding acquisition, Methodology, Project administration, Resources, Supervision, Validation, Writing - review & editing.

## Declaration of Competing Interest

We declare that none of the authors have competing financial or non-financial interests.

## Appendix Supplementary materials

Image, application 1
