# Supplementary material for: T2 heterogeneity as an in vivo marker of microstructural integrity in medial temporal lobe subfields in ageing and mild cognitive impairment
Source: Neuroimage. 2021 Sep;238:118214. doi: 10.1016/j.neuroimage.2021.118214 (PMC8350145; doi:10.1016/j.neuroimage.2021.118214)
Supplement: Supplementary file 1 [file mmc1.docx]

# **Supplementary information**

# T2 heterogeneity as an *in vivo* marker of microstructural integrity in medial temporal lobe subfields in ageing and mild cognitive impairment

## Individual study cohort information

Demographic information, neuropsychological test data and data from MRI summaries for study 1 and study 2 can be found in Supplementary Tables 1 and 2, respectively.

Supplementary Table 1 | Study 1 cohort information.

Demographic, neuropsychology, and MRI structural measure info for study 1 cohort. Data show mean ± standard deviation. Some neuropsychological measures are missing for a small number of participants. The numbers, and which groups they belong to, is indicated in the leftmost column. All volumes shown have been corrected for intracranial volume. HC = Healthy Control; MCI = Mild Cognitive Impairment; YOE = years of education; MoCA = Montreal Cognitive Assessment;; PAL = CANTAB Paired Associate Learning

| **Demographics** | HC | MCI |
| --- | --- | --- |
| N (male: female) | 50 (21:29) | 30 (14:16) |
| Age (years) | 67.7 ± 9.03 | 70.7 ± 8.55 |
| YOE | 15.6 ± 2.66 | 14.5 ± 2.70 |
|  |  |  |
| **Neuropsychological testing** |  |  |
| MoCA (/30) | 28.0 ± 1.32 | 23.0 ± 2.83 |
| PAL (-2 MCI) |  |  |
| Accuracy | .706 ± .131 | .534 ± .145 |
| Mean Reaction Time (ms) | 2120 ± 488 | 2960 ± 1490 |
|  |  |  |
| **MRI structural measures** |  |  |
| Right DG |  |  |
| T2σ | 0.08 ± 0.02 | 0.09 ± 0.02 |
| T2μ | 4.63 ± 0.03 | 4.63 ± 0.03 |
| Volume | 0.56 ± 0.07 | 0.50 ± 0.08 |
| Left DG |  |  |
| T2σ | 0.09 ± 0.02 | 0.09 ± 0.02 |
| T2μ | 4.63 ± 0.03 | 4.63 ± 0.02 |
| Volume | 0.54 ± 0.07 | 0.49 ± 0.08 |
| Right CA |  |  |
| T2σ | 0.09 ± 0.02 | 0.10 ± 0.02 |
| T2μ | 4.60 ± 0.03 | 4.60 ± 0.03 |
| Volume | 0.93 ± 0.13 | 0.80 ± 0.16 |
| Left CA |  |  |
| T2σ | 0.09 ± 0.02 | 0.10 ± 0.02 |
| T2μ | 4.61 ± 0.03 | 4.60 ± 0.02 |
| Volume | 0.91 ± 0.12 | 0.80 ± 0.15 |
| Right SUB |  |  |
| T2σ | 0.10 ± 0.02 | 0.12 ± 0.03 |
| T2μ | 4.48 ± 0.04 | 4.44 ± 0.04 |
| Volume | 0.24 ± 0.03 | 0.22 ± 0.04 |
| Left SUB |  |  |
| T2σ | 0.10 ± 0.02 | 0.12 ± 0.02 |
| T2μ | 4.48 ± 0.05 | 4.43 ± 0.06 |
| Volume | 0.25 ± 0.04 | 0.23 ± 0.05 |
| Right EC |  |  |
| T2σ | 0.10 ± 0.02 | 0.11 ± 0.02 |
| T2μ | 4.53 ± 0.04 | 4.52 ± 0.04 |
| Volume | 0.27 ± 0.05 | 0.24 ± 0.05 |
| Left EC |  |  |
| T2σ | 0.10 ± 0.02 | 0.12 ± 0.02 |
| T2μ | 4.52 ± 0.05 | 4.50 ± 0.04 |
| Volume | 0.31 ± 0.04 | 0.29 ± 0.07 |
| Right BA35 |  |  |
| T2σ | 0.09 ± 0.02 | 0.10 ± 0.02 |
| T2μ | 4.55 ± 0.04 | 4.56 ± 0.03 |
| Volume | 0.27 ± 0.06 | 0.25 ± 0.06 |
| Left BA35 |  |  |
| T2σ | 0.09 ± 0.02 | 0.10 ± 0.02 |
| T2μ | 4.55 ± 0.03 | 4.54 ± 0.03 |
| Volume | 0.26 ± 0.05 | 0.25 ± 0.07 |

Supplementary Table 2 | Study 2 cohort information.

Demographic, neuropsychology, and MRI structural measure info for study 2 cohort. Data show mean ± standard deviation. Some neuropsychological measures are missing for a small number of participants. The numbers, and which groups they belong to, is indicated in the leftmost column. All volumes shown have been corrected for intracranial volume. HC = Healthy Control; MCI = Mild Cognitive Impairment; YOE = years of education; ACE-III = Addenbrookes Cognitive Examination-III; PAL = CANTAB Paired Associate Learning.

| **Demographics** | HC | MCI |
| --- | --- | --- |
| N (male: female) | 49 (26:23) | 19 (13:6) |
| Age | 70.8 ± 7.79 | 74.5 ± 9.49 |
| YOE | 16.0 ± 3.57 | 13.7 ± 3.00 |
|  |  |  |
| **Neuropsychological testing** |  |  |
| ACE-III /100 | 94.9 ± 3.17 | 80.2 ± 6.62 |
| PAL (-2 HC, -4 MCI) |  |  |
| Accuracy | .695 ± .113 | .493 ± .184 |
| Mean Reaction Time (ms) | 2280 ± 485 | 3880 ± 2350 |
|  |  |  |
| **MRI structural measures** |  |  |
| Right DG |  |  |
| T2σ | 0.10 ± 0.01 | 0.11 ± 0.01 |
| T2μ | 4.71 ± 0.03 | 4.72 ± 0.03 |
| Volume | 0.48 ± 0.06 | 0.42 ± 0.08 |
| Left DG |  |  |
| T2σ | 0.11 ± 0.01 | 0.12 ± 0.01 |
| T2μ | 4.70 ± 0.03 | 4.70 ± 0.04 |
| Volume | 0.46 ± 0.06 | 0.39 ± 0.09 |
| Right CA |  |  |
| T2σ | 0.11 ± 0.01 | 0.12 ± 0.01 |
| T2μ | 4.68 ± 0.03 | 4.68 ± 0.03 |
| Volume | 0.79 ± 0.09 | 0.65 ± 0.15 |
| Left CA |  |  |
| T2σ | 0.12 ± 0.01 | 0.12 ± 0.01 |
| T2μ | 4.67 ± 0.03 | 4.67 ± 0.05 |
| Volume | 0.79 ± 0.10 | 0.62 ± 0.15 |
| Right SUB |  |  |
| T2σ | 0.14 ± 0.01 | 0.16 ± 0.02 |
| T2μ | 4.50 ± 0.05 | 4.47 ± 0.06 |
| Volume | 0.22 ± 0.02 | 0.18 ± 0.04 |
| Left SUB |  |  |
| T2σ | 0.15 ± 0.01 | 0.17 ± 0.02 |
| T2μ | 4.51 ± 0.04 | 4.47 ± 0.05 |
| Volume | 0.23 ± 0.03 | 0.18 ± 0.04 |
| Right EC |  |  |
| T2σ | 0.13 ± 0.01 | 0.15 ± 0.02 |
| T2μ | 4.58 ± 0.04 | 4.54 ± 0.05 |
| Volume | 0.26 ± 0.04 | 0.21 ± 0.06 |
| Left EC |  |  |
| T2σ | 0.14 ± 0.01 | 0.17 ± 0.02 |
| T2μ | 4.57 ± 0.04 | 4.54 ± 0.06 |
| Volume | 0.30 ± 0.04 | 0.26 ± 0.06 |
| Right BA35 |  |  |
| T2σ | 0.11 ± 0.01 | 0.13 ± 0.02 |
| T2μ | 4.65 ± 0.03 | 4.65 ± 0.08 |
| Volume | 0.24 ± 0.05 | 0.17 ± 0.05 |
| Left BA35 |  |  |
| T2σ | 0.12 ± 0.02 | 0.13 ± 0.02 |
| T2μ | 4.63 ± 0.04 | 4.61 ± 0.06 |
| Volume | 0.24 ± 0.05 | 0.20 ± 0.05 |

## T2 variation between subfields

### Results

#### T2 heterogeneity

The mixed model analysis revealed a significant main effect of subfield (F(4,576)=238, p<.0001) on T2 heterogeneity across different diagnoses in our mixed model analysis. The lowest T2 heterogeneity was observed in DG. CA and BA35 were significantly more heterogenous than DG (p_corr_ <.0001), but not different from each other (p_corr_ =1.00). EC was the next most heterogenous (p_corr_ <.0001 vs CA123), and SUB had the greatest T2 heterogeneity of all subfields (p_corr_ =.024 vs EC). We also observed a significant main effect of hemisphere (Left>Right, F(1,144)=18.6, p<.0001) as well as an interaction between subfield and hemisphere (F(4,576)=3.12, p=.014). Follow-up analysis of this interaction using two-way ANOVA with Bonferroni multiple comparisons test reveals significantly greater T2 heterogeneity selectively in DG in the left hemisphere compared to the right (p_corr_ =.028) – but all other subfields did not differ between hemispheres.

#### Absolute T2

We also compared absolute T2 between subfields. Our mixed model analysis revealed a substantial overall difference in T2μ between subfields (F(4,576)=1220, p<.0001). DG had the highest T2μ, followed by CA, BA35, EC and finally SUB. All subfields were significantly different from each other at the p_corr_<.0001 level. We also observed a significant main effect of hemisphere (Right>Left, F(1,144)=15.1, p=.0002), and a subfield*hemisphere interaction (F(4,576)=7.15, p<.0001). Follow-up analysis of this interaction using two-way ANOVA with Bonferroni multiple comparisons test reveals a significantly higher T2μ in left EC (p_corr_ =.008) and right BA35 (p_corr_ <.0001).

### Discussion

We show several differences between absolute T2 in subfields of the MTL including a relatively prolonged T2 in the dentate gyrus compared to all other subfields, followed closely by CA1-3, and a relatively short T2 in the subiculum. This could reflect different cell types and microstructural features of each subfield, such as myelination. Our findings are highly in line with previous studies of quantitative MRI (qMRI) proxies of myelination, including quantitative T1 (Marques and Gruetter, 2013) and T1w/T2w ratio (DeKraker et al., 2018, de Wael et al., 2018), whereby subiculum is consistently found to have high qMRI-estimated levels of myelination compared to lower levels in DG, with CA1-3 in between. DG may also be a subfield more susceptible to myelin loss in ageing, leading to the relatively high T2 seen in this subfield. This is supported by Radhakrishnan et al. (2020) who show that an MRI proxy for myelination in DG/CA3 is the best correlate of behaviour across all subfields. Furthermore, as DG is one of the last regions to develop myelin during development (Abrahám et al., 2010), it is supportive of the retrogenesis theory, which states that the brain degrades in an order in reverse to that in which it develops in AD (Reisberg et al., 2002). Even in a cohort of cognitively healthy older controls around 20% could be expected to harbour incipient AD pathology (Kern et al., 2018), explaining the relatively elevated T2 in DG.

An alternative candidate for driving T2 changes is localised non-haem iron deposits which decrease T2 and have been shown to be more prevalent in the subiculum, and less so in DG (Antharam et al., 2012, Morris et al., 1994). Contrary to this, a review by Neely et al. (2019) shows how, at least in the rodent hippocampus, DG tends to have relatively high iron content, suggesting that iron may not be the primary driver of T2 differences between subfields.

In a recent study of magnetic resonance elastography in hippocampal subfields, Delgorio et al. (2021) found that subiculum and entorhinal cortex had a relatively low damping ratio (a measure of physical integrity), compared to CA and DG regions. This is in line with our finding of the greatest T2 heterogeneity in these same subfields. T2 heterogeneity and tissue elastography may well be sensitive to some of the same features, and future studies should aim to directly compare the two measures, their clinical utility, and whether they offer any combined benefit for understanding hippocampal microstructure.

The two known studies that have observed quantitative T2 in hippocampal subfields *in vitro* both found little difference between the subfields in absolute T2 (Huesgen et al., 1993, Antharam et al., 2012). This is in contrast to our findings, which may be due to *in vitro* vs *in vivo* methodological differences. T2 is also likely to vary throughout individual cell layers of allo- and neocortex given different density of cell bodies, proportions of glial cells and, as mentioned previously, densities of non-haem iron. Such differences in individual cortical layers within each subfield are lost here due to limitations in image resolution and contrast. Further exploration of these layers using ultra high-field MRI would be an interesting avenue of future research.

We have previously made the argument that any conclusions on the causes of changes in absolute T2 are difficult to make given the myriad different factors that affect T2 in opposing ways. We see that subfields with lower absolute T2 values have greater T2 heterogeneity. This suggests that the increases in T2 heterogeneity are brought about by a larger proportion of hypointense regions of T2 (T2-decreasing factors such as iron or myelin). However, when considering the effects of pathology, absolute T2 and T2 heterogeneity show very different patterns.

## Supplementary path analysis statistics

Supplementary Table 3 shows data summarised in Figure 4 of the main manuscript. Not all values could be shown on the figure for clarity, and so are presented here.

Supplementary Table 3 | Summary statistics of direct paths in HC path analysis

Statistically significant p values are marked with asterisks (*). p-values marked with † indicate statistical tests that do not survive FDR correction for multiple comparisons (p<0.02).

| Path | | Standardized Regression Weight | | P-value |
| --- | --- | --- | --- | --- |
| Age 🡪 PAL Reaction Time | | | .274 | *.007 |
| Age 🡪 PAL Total Accuracy | | | -.325 | <.001 |
| Age 🡪 [Subfield] T2 heterogeneity | | |  |  |
|  | DG | | .315 | *.001 |
|  | CA | | .338 | *<.001 |
|  | SUB | | .298 | *.002 |
|  | EC | | .329 | *<.001 |
|  | BA35 | | .400 | *<.001 |
| Age 🡪 [Subfield] Volume | | |  |  |
|  | DG | | -.277 | *.005 |
|  | CA | | -.347 | *<.001 |
|  | SUB | | -.214 | †.032 |
|  | EC | | -.197 | .052 |
|  | BA35 | | -.153 | .150 |
| [Subfield] T2 heterogeneity 🡪 [Subfield] Volume | | |  |  |
|  | DG | | -.199 | †.028 |
|  | CA | | -.220 | *.006 |
|  | SUB | | -.208 | *.016 |
|  | EC | | -.220 | *.017 |
|  | BA35 | | -.191 | .065 |
| [Subfield] T2 heterogeneity 🡪 PAL | | |  |  |
| PAL Reaction Time | DG | | .310 | .273 |
|  | CA | | -.514 | †.040 |
|  | SUB | | -.016 | .932 |
|  | EC | | -.126 | .447 |
|  | BA35 | | -.035 | .841 |
|  | | |  |  |
| PAL Total Accuracy | DG | | -.390 | .154 |
|  | CA | | .174 | .473 |
|  | SUB | | -.266 | .148 |
|  | EC | | .254 | .113 |
|  | BA35 | | .274 | .102 |
| [Subfield] Volume 🡪 PAL | | |  |  |
| PAL Reaction Time | DG | | .189 | .127 |
|  | CA | | -.557 | *<.001 |
|  | SUB | | .159 | .209 |
|  | EC | | .052 | .637 |
|  | BA35 | | -.092 | .342 |
|  | | |  |  |
| PAL Total Accuracy | DG | | -.232 | .053 |
|  | CA | | .193 | .195 |
|  | SUB | | .140 | .251 |
|  | EC | | -.058 | .585 |
|  | BA35 | | .283 | *.002 |

### MCI Path Analysis


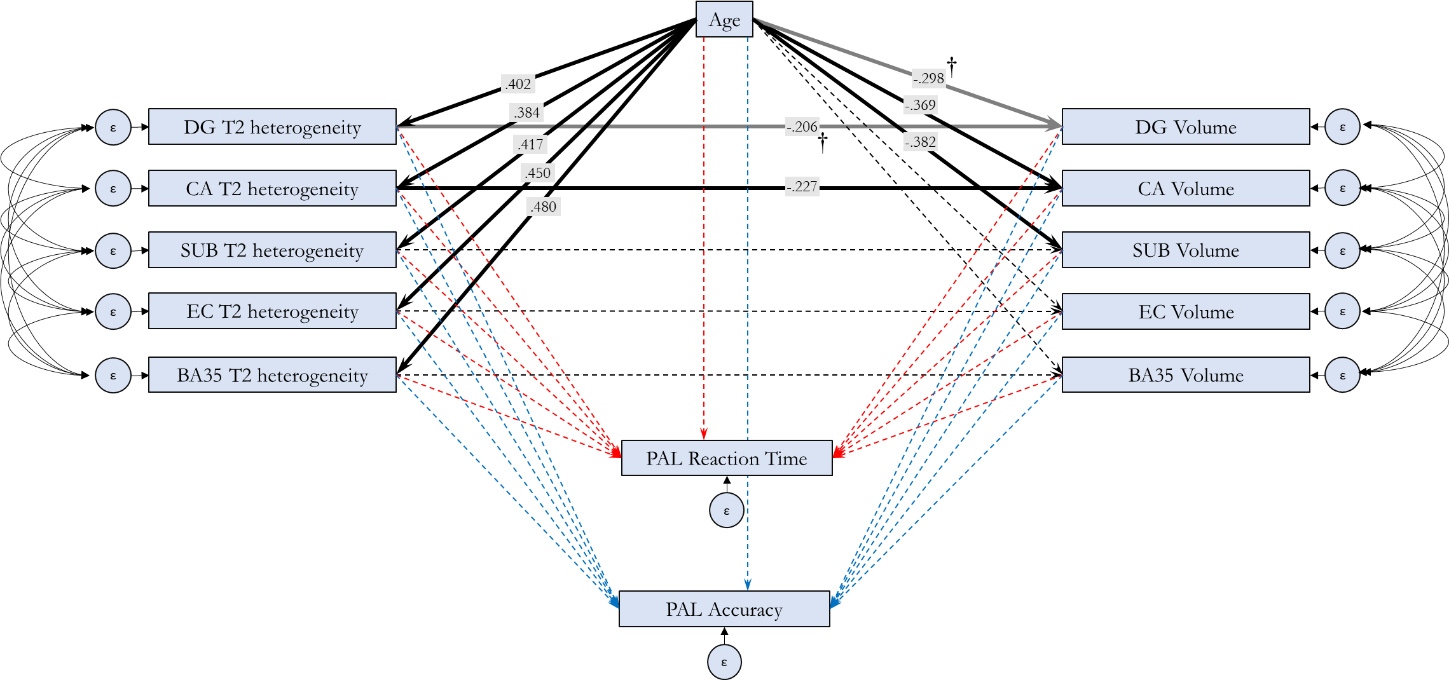


Supplementary Figure 1 | Path analysis showing the relationship between Age, T2 heterogeneity, volume and memory in MTL subfields in people with MCI.

Bold arrows represent statistically significant relationships, with standardized B values indicated in overlaid boxes. Two models were run, each assessing one outcome measure of the PAL task. Black lines represent paths shared between the models. Unique paths to each model are shown in red (PAL reaction time as dependent variable) and blue (PAL Total Accuracy as dependent variable). Curves lines represent error term covariances defined in the model. Paths faded and marked with † indicate statistical tests that do not survival FDR correction for multiple comparisons (p<0.011). All subfield volumes were normalised to ICV prior to entering into the model.

Model fit for MCI path analysis was poorer than that for healthy older controls (*χ^2^*=34.7, df = 20, p=.021; C_min_/DF=1.74; GFI=.895, AGFI=.592; CFI=.968; RMSEA=.133 ± 90% CI [.051-.205]; p_close_^=^.048).

Supplementary Table 4 | Summary statistics of direct paths in MCI path analysis

Statistically significant p values are marked with asterisks (*). p-values marked with † indicate statistical tests that do not survival FDR correction for multiple comparisons (p<0.011)

| Path | | Standardized Regression Weight | | P-value |
| --- | --- | --- | --- | --- |
| Age 🡪 PAL Reaction Time | | | .029 | .870 |
| Age 🡪 PAL Total Accuracy | | | -.029 | .856 |
| Age 🡪 [Subfield] T2 heterogeneity | | |  |  |
|  | DG | | .402 | *.004 |
|  | CA | | .384 | *.007 |
|  | SUB | | .417 | *.003 |
|  | EC | | .450 | *.001 |
|  | BA35 | | .480 | <.001 |
| Age 🡪 [Subfield] Volume | | |  |  |
|  | DG | | -.298 | †.040 |
|  | CA | | -.369 | *.007 |
|  | SUB | | -.382 | *.008 |
|  | EC | | -.256 | .090 |
|  | BA35 | | .007 | .964 |
| [Subfield] T2 heterogeneity 🡪 [Subfield] Volume | | |  |  |
|  | DG | | -.206 | †.040 |
|  | CA | | -.227 | *.004 |
|  | SUB | | -.095 | .299 |
|  | EC | | -.198 | .081 |
|  | BA35 | | -.140 | .269 |
| [Subfield] T2 heterogeneity 🡪 PAL | | |  |  |
| PAL Reaction Time | DG | | -.475 | .171 |
|  | CA | | -.096 | .781 |
|  | SUB | | .092 | .735 |
|  | EC | | .178 | .503 |
|  | BA35 | | .492 | .130 |
|  | | |  |  |
| PAL Total Accuracy | DG | | .256 | .454 |
|  | CA | | -.143 | .724 |
|  | SUB | | .054 | .848 |
|  | EC | | -.481 | .098 |
|  | BA35 | | .205 | .542 |
| [Subfield] Volume 🡪 PAL | | |  |  |
| PAL Reaction Time | DG | | -.040 | .934 |
|  | CA | | -.410 | .339 |
|  | SUB | | .274 | .329 |
|  | EC | | -.016 | .769 |
|  | BA35 | | -.041 | .965 |
|  | | |  |  |
| PAL Total Accuracy | DG | | .292 | .336 |
|  | CA | | -.252 | .541 |
|  | SUB | | .315 | .247 |
|  | EC | | .277 | .173 |
|  | BA35 | | -.130 | .471 |

### ‘Reversed’ Path Analysis (volume predicts T2 heterogeneity)


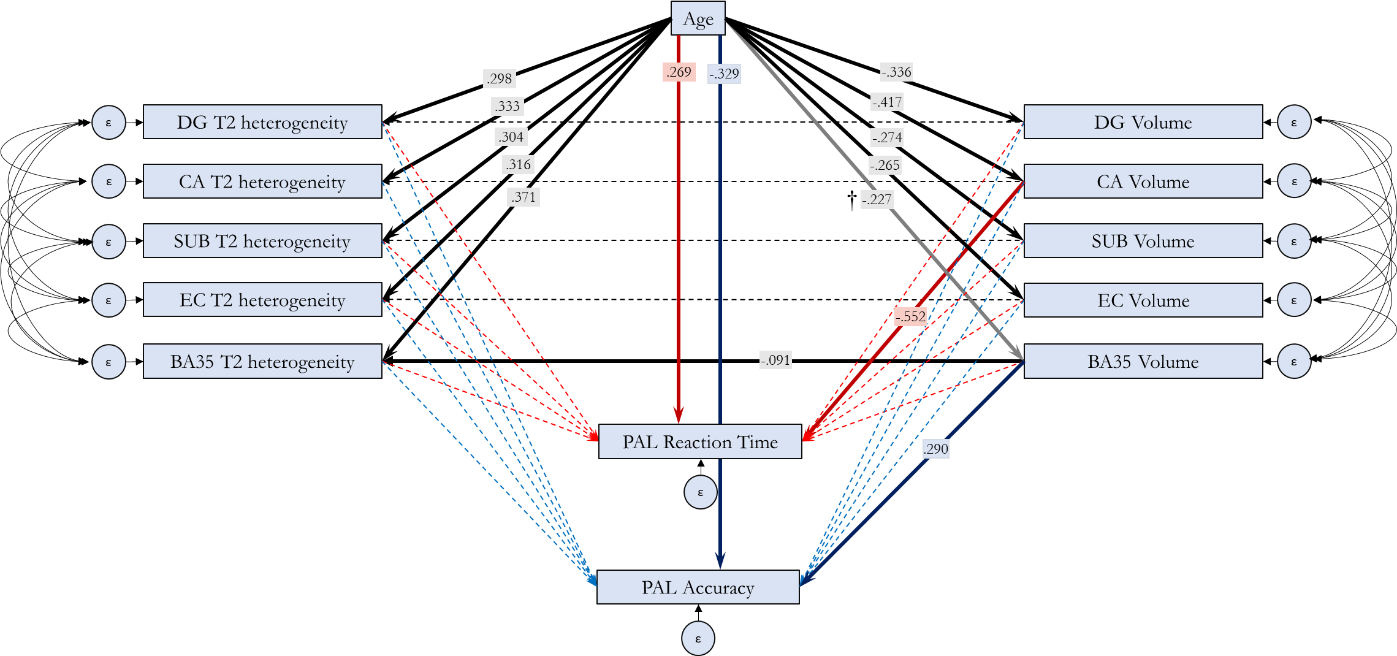


Supplementary Figure 2 | ‘Reversed’ path analysis showing the relationship between Age, T2 heterogeneity, volume and memory in MTL subfields in HC cohort, with volume allowed to predict T2 heterogeneity.

Bold arrows represent statistically significant relationships, with standardized B values indicated in overlaid boxes. Two models were run, each assessing one outcome measure of the PAL task. Black lines represent paths shared between the models. Unique paths to each model are shown in red (PAL reaction time as dependent variable) and blue (PAL Total Accuracy as dependent variable). Curves lines represent error term covariances defined in the model. Paths marked with † indicate statistical tests that do not survival FDR correction for multiple comparisons (p<0.019). All subfield volumes were normalised to ICV prior to entering into the model.

Model fit for MCI path analysis was poorer than that for healthy older controls (*χ^2^*=32.3, df = 20, p=.040; C_min_/DF=1.62; GFI=.950, AGFI=.803; CFI=.984; RMSEA=.080 ± 90% CI [.017-.130]; p_close_^=^.159).

Supplementary Table 5 | Summary statistics of direct paths in reversed path analysis

This analysis is identical to the model presented in the main manuscript, and supplementary table 3, except that volumes were allowed predict T2 heterogeneity for each subfield, instead of vice versa. Statistically significant p values are marked with asterisks (*). p-values marked with † indicate statistical tests that do not survival FDR correction for multiple comparisons (p<0.019)

| Path | | Standardized Regression Weight | | P-value |
| --- | --- | --- | --- | --- |
| Age 🡪 PAL Reaction Time | | | .269 | *.008 |
| Age 🡪 PAL Total Accuracy | | | -.329 | *.001 |
| Age 🡪 [Subfield] T2 heterogeneity | | |  |  |
|  | DG | | .298 | *.002 |
|  | CA | | .333 | *<.001 |
|  | SUB | | .304 | *.002 |
|  | EC | | .316 | *.001 |
|  | BA35 | | .371 | *<.001 |
| Age 🡪 [Subfield] Volume | | |  |  |
|  | DG | | -.336 | *<.001 |
|  | CA | | -.417 | *<.001 |
|  | SUB | | -.274 | *.005 |
|  | EC | | -.265 | *.007 |
|  | BA35 | | -.227 | †.023 |
| [Subfield] Volume 🡪 [Subfield] T2 heterogeneity | | |  |  |
|  | DG | | -.062 | .111 |
|  | CA | | -.015 | .750 |
|  | SUB | | -.030 | .554 |
|  | EC | | -.064 | .277 |
|  | BA35 | | -.091 | *.006 |
| [Subfield] T2 heterogeneity 🡪 PAL | | |  |  |
| PAL Reaction Time | DG | | .300 | .279 |
|  | CA | | -.502 | †.035 |
|  | SUB | | -.016 | .932 |
|  | EC | | -.122 | .440 |
|  | BA35 | | -.034 | .844 |
|  | | |  |  |
| PAL Total Accuracy | DG | | -.391 | .158 |
|  | CA | | .175 | .463 |
|  | SUB | | -.271 | .150 |
|  | EC | | .253 | .108 |
|  | BA35 | | .275 | .110 |
| [Subfield] Volume 🡪 PAL | | |  |  |
| PAL Reaction Time | DG | | .187 | .132 |
|  | CA | | -.552 | *<.001 |
|  | SUB | | .156 | .213 |
|  | EC | | .051 | .640 |
|  | BA35 | | -.091 | .351 |
|  | | |  |  |
| PAL Total Accuracy | DG | | -.238 | .056 |
|  | CA | | .197 | .195 |
|  | SUB | | .143 | .254 |
|  | EC | | -.059 | .589 |
|  | BA35 | | .290 | *.003 |

### Path Analysis Covariance Matrices

Supplementary Table 6 | Covariance estimates for error terms within Path Analysis model for healthy older controls.
Values above the midline (yellow) represent covariance values between error terms on subfield volumes. Values below the midline (blue) represent covariance values between error terms on subfield T2σ (T2 heterogeneity).

|  | DG | | CA123 | | SUB | | EC | | BA35 | |
| --- | --- | --- | --- | --- | --- | --- | --- | --- | --- | --- |
|  | B ± SE | P | B ± SE | P | B ± SE | P | B ± SE | P | B ± SE | P |
| DG | - | - | .427 ± .081 | <.001 | .273 ± .076 | <.001 | .198 ± .071 | 0.005 | .162 ± .073 | 0.027 |
| CA123 | .730 ± .111 | <.001 | - | - | .425 ± .082 | <.001 | .273 ± .071 | <.001 | .167 ± .071 | 0.19 |
| SUB | .669 ± .106 | <.001 | .635 ± .104 | <.001 | - | - | .322 ± .076 | <.001 | .185 ± .074 | 0.012 |
| EC | .608 ± .101 | <.001 | .555 ± .098 | <.001 | .595 ± .100 | <.001 | - | - | .184 ± .071 | 0.01 |
| BA35 | .591 ± .098 | <.001 | .588 ± .098 | <.001 | .553 ± .095 | <.001 | .549 ± .094 | <.001 | - | - |

Supplementary Table 7 | Covariance estimates for error terms within Path Analysis model for people with MCI.
Values above the midline (yellow) represent covariance values between error terms on subfield volumes. Values below the midline (blue) represent covariance values between error terms on subfield T2σ (T2 heterogeneity).

|  | DG | | CA123 | | SUB | | EC | | BA35 | |
| --- | --- | --- | --- | --- | --- | --- | --- | --- | --- | --- |
|  | B ± SE | P | B ± SE | P | B ± SE | P | B ± SE | P | B ± SE | P |
| DG | - | - | 1.01 ± .236 | <.001 | .859 ± .236 | <.001 | .654 ± .217 | 0.003 | .553 ± .177 | 0.002 |
| CA123 | .860 ± .201 | <.001 | - | - | 1.09 ± .263 | <.001 | .852 ± .239 | <.001 | .724 ± .196 | <.001 |
| SUB | .852 ± .219 | <.001 | .735 ± .188 | <.001 | - | - | 1.07 ± .276 | <.001 | .802 ± .216 | <.001 |
| EC | .835 ± .260 | 0.001 | .810 ± .230 | <.001 | 1.08 ± .279 | <.001 | - | - | .742 ± .209 | <.001 |
| BA35 | 1.01 ± .248 | <.001 | .868 ± .213 | <.001 | .944 ± .241 | <.001 | 1.23 ± .312 | <.001 | - | - |

## The relationship between MTL T2 heterogeneity and volume

We ran partial correlations to explore the relationship between absolute volume (uncorrected for intracranial volume) and T2 heterogeneity, correcting for age. The results of this analysis are presented in Supplementary Table 8.

Supplementary Table 8 | Partial correlation statistics of T2 heterogeneity in MTL subfields vs volumes corrected and uncorrected for ICV.

Asterisks represent significant p-values after FDR-correction for multiple comparisons. Partial correlations correct for the effect of age. ‘Raw’ volume indicated volume uncorrected for intracranial volume (ICV).

|  |  | T2 heterogeneity | |
| --- | --- | --- | --- |
|  |  | r | p-value |
| Raw Volume | DG | -.177 | .081 |
|  | CA | -.234 | .020 |
|  | SUB | -.005 | .965 |
|  | EC | -.065 | .527 |
|  | BA35 | -.095 | .350 |
|  |  |  |  |
| ICV-corrected | DG | -.354 | *<.001 |
|  | CA | -.277 | *.006 |
|  | SUB | -.342 | *.001 |
|  | EC | -.339 | *.001 |
|  | BA35 | -.181 | .075 |

From this analysis, we do not see evidence that the absolute (uncorrected) volume of a subfield is systematically associated with T2σ. As T2σ tends to be highly associated with volume corrected for intracranial volume (a measure which estimates premorbid MTL volume, and therefore represents something closer to ‘volume change from healthy state’), we argue that this supports T2σ as a clinically relevant measure of microstructural change, distinct from volumetry. Furthermore, it supports the use of automated hippocampal masking procedures as a means of measuring T2 heterogeneity.

## SNR calculation

We compared signal to noise ratio of HC and MCI groups to ensure differences in T2 heterogeneity were not due to greater noise in scans of people with MCI. Signal was measured from bilateral hippocampal ROIs and noise from surrounding air on second (middle) echo of TSE image. SNR was multiplied by 0.66 Raleigh distribution correction factor. Healthy controls possessed an average SNR of 45.4±14.7 compared to 40.7±11.7 in MCI. An unpaired t-test revealed no significant difference between groups (t=0.840, p=.411).

ABRAHÁM, H., VINCZE, A., JEWGENOW, I., VESZPRÉMI, B., KRAVJÁK, A., GÖMÖRI, E. & SERESS, L. 2010. Myelination in the human hippocampal formation from midgestation to adulthood. *International journal of developmental neuroscience : the official journal of the International Society for Developmental Neuroscience,* 28**,** 401-10.

ANTHARAM, V., COLLINGWOOD, J. F., BULLIVANT, J.-P., DAVIDSON, M. R., CHANDRA, S., MIKHAYLOVA, A., FINNEGAN, M. E., BATICH, C., FORDER, J. R. & DOBSON, J. 2012. High field magnetic resonance microscopy of the human hippocampus in Alzheimer's disease: Quantitative imaging and correlation with iron. *NeuroImage,* 59**,** 1249-1260.

DE WAEL, R. V., LARIVIÈRE, S., CALDAIROU, B., HONG, S.-J., MARGULIES, D. S., JEFFERIES, E., BERNASCONI, A., SMALLWOOD, J., BERNASCONI, N. & BERNHARDT, B. C. 2018. Anatomical and microstructural determinants of hippocampal subfield functional connectome embedding. *Proceedings of the National Academy of Sciences,* 115**,** 201803667.

DEKRAKER, J., FERKO, K. M., LAU, J. C., KÖHLER, S. & KHAN, A. R. 2018. Unfolding the hippocampus: An intrinsic coordinate system for subfield segmentations and quantitative mapping. *NeuroImage,* 167**,** 408-418.

DELGORIO, P. L., HISCOX, L. V., DAUGHERTY, A. M., SANJANA, F., POHLIG, R. T., ELLISON, J. M., MARTENS, C. R., SCHWARB, H., MCGARRY, M. D. J. & JOHNSON, C. L. 2021. Effect of Aging on the Viscoelastic Properties of Hippocampal Subfields Assessed with High-Resolution MR Elastography. *Cerebral Cortex*.

HUESGEN, C. T., BURGER, P. C., CRAIN, B. J. & JOHNSON, G. A. 1993. In vitro MR microscopy of the hippocampus in Alzheimer's disease. *Neurology,* 43**,** 145-145.

KERN, S., ZETTERBERG, H., KERN, J., ZETTERGREN, A., WAERN, M., HÖGLUND, K., ANDREASSON, U., WETTERBERG, H., BÖRJESSON-HANSON, A., BLENNOW, K. & SKOOG, I. 2018. Prevalence of preclinical Alzheimer disease: Comparison of current classification systems. *Neurology,* 90**,** e1682-e1691.

MARQUES, J. P. & GRUETTER, R. 2013. New Developments and Applications of the MP2RAGE Sequence - Focusing the Contrast and High Spatial Resolution R1 Mapping. *PLoS ONE,* 8**,** e69294.

MORRIS, C. M., KERWIN, J. M. & EDWARDSON, J. A. 1994. Non-haem iron histochemistry of the normal and Alzheimer's disease hippocampus. *Neurodegeneration : a journal for neurodegenerative disorders, neuroprotection, and neuroregeneration,* 3**,** 267-75.

NEELY, C., LIPPI, S., LANZIROTTI, A. & FLINN, J. 2019. Localization of Free and Bound Metal Species through X-Ray Synchrotron Fluorescence Microscopy in the Rodent Brain and Their Relation to Behavior. *Brain Sciences,* 9**,** 74.

RADHAKRISHNAN, H., STARK, S. M. & STARK, C. E. L. 2020. Microstructural Alterations in Hippocampal Subfields Mediate Age-Related Memory Decline in Humans. *Frontiers in Aging Neuroscience,* 12**,** 94.

REISBERG, B., FRANSSEN, E. H., SOUREN, L. E. M., AUER, S. R., AKRAM, I. & KENOWSKY, S. 2002. Evidence and mechanisms of retrogenesis in Alzheimer's and other dementias: Management and treatment import. *American Journal of Alzheimer's Disease & Other Dementiasr,* 17**,** 202-212.
